# An Exploration of the Factor Structure of Executive Functioning in Children

**DOI:** 10.3389/fpsyg.2018.01179

**Published:** 2018-07-13

**Authors:** David Messer, Marialivia Bernardi, Nicola Botting, Elisabeth L. Hill, Gilly Nash, Hayley C. Leonard, Lucy A. Henry

**Affiliations:** ^1^Centre for Research in Education and Educational Technology, The Open University, Milton Keynes, United Kingdom; ^2^Language and Communication Science, City University of London, London, United Kingdom; ^3^Department of Psychology, Goldsmiths, University of London, London, United Kingdom; ^4^School of Psychology, University of Surrey, Guildford, United Kingdom

**Keywords:** executive functioning, children, factor structure, task impurity, unity and diversity

## Abstract

There has been considerable debate and interest in the factor structure of executive functioning (EF). For children and young people, there is evidence of a progression from a single factor to a more differentiated structure, although the precise nature of these factors differs between investigations. The purpose of the current study was to look at this issue again with another sample, and try to understand possible reasons for previous differences between investigations. In addition, we examined the relationship between less central EF tasks, such as fluency and planning, to the more common tasks of updating/executive working memory (EWM), inhibition, and switching/shifting. A final aim was to carry out analyses which are relevant to the debate about whether EF is influenced by language ability, or language ability is influenced by EF. We reasoned that if language ability affects EF, a factor analysis of verbal and non-verbal EF tasks might result in the identification of a factor which predominantly contains verbal tasks and a factor that predominately contains non-verbal tasks. Our investigation involved 128 typically developing participants (mean age 10:4) who were given EF assessments that included verbal and non-verbal versions of each task: EWM; switching; inhibition; fluency; and planning. Exploratory factor analyses on EWM, switching, and inhibition produced a structure consisting of inhibition in one factor and the remaining tasks in another. It was decided to exclude verbal planning from the next analyses of all the ten tasks because of statistical considerations. Analysis of the remaining nine EF tasks produced two factors, one factor containing the two inhibition tasks, and another factor that contained all the other tasks (switching, EWM, fluency, and non-verbal planning). There was little evidence that the verbal or non-verbal elements in these tasks affected the factor structure. Both these issues are considered in the discussion, where there is a general evaluation of findings about the factor structure of EF.

## Introduction

Executive functioning (EF) continues to be an important topic of research in relation to children and young people (Diamond, 2013). There is a growing consensus about the cognitive processes and relevant assessment procedures for the investigation of EF. However, there has been a longstanding discussion about whether the different forms of EF should be considered as making up one single area of cognitive functioning or involve separable/distinct statistical factors, as well as discussion about the nature of, and relationships between, identifiable factors. Such investigations can help with the understanding of relationships between different tasks that are used to assess EF. These are important and challenging issues similar to those seen in research on the separability of intelligence into different factors (McGrew, 2005).

### The Structure of EF and Its Development

Research with adults tends to identify three EF factors (inhibition, switching, and updating), which are related to each other, but nevertheless are separable, hence the suggestion that EF involves both unity and diversity (Miyake et al., 2000). In relation to children and young people, there is a widely held view that with increasing age the elements of EF become more separable from one another, although there are disagreements about which factors are separable and at which ages. We use the term “factor” to refer to EF tasks that have been identified on a statistical basis as being related to one another. “Component” is used to refer to the three commonly identified forms of EF, specifically updating/executive working memory (EWM; which involves the executive component of working memory), switching/shifting, and inhibition. For children between 3 and 6 years, several investigators (Wiebe et al., 2008, 2011; Hughes et al., 2010) have reported that EF is best described as a single factor. Thus, it appears that in the pre-school age, EF may be undifferentiated and does not involve statistically separable factors, so that individual differences (i.e., the differences between children) across different EF components appear to be influenced by a general cognitive capacity such as attention (Garon et al., 2008).

In the 6 to 12 year age range a number of different factor structures have been identified. For children aged 7–9 years and 10–11 years, Xu et al. (2013) compared five models of the structure of EF, reporting that a one-factor model was reasonably good at accounting for their data (inhibition, EWM, and switching). However, several groups of researchers have identified two-factor models of EF in the 6–12 years age range, although the models differ with regards to which EF tasks occur in the same factor. At 9–12 years, van der Sluis et al. (2007) reported that EWM and shifting were separate factors, but a separate inhibition factor was not supported by their data. In another study with 11–12-year-old children, St Clair-Thompson and Gathercole (2006) identified updating/EWM and inhibition as separate factors, but not switching. van der Ven et al. (2013) also reported a two-factor model (an updating factor and a combined inhibition and shifting factor), but noted that verbal ability and motor speed were additionally implicated. Finally, Huizinga et al. (2006) found good evidence for two factors (EWM, set shifting) in 7- and 11-year-olds (and also in 15- and 21-year-olds), although there was no evidence for an underlying inhibition construct as the three inhibition measures they used did not relate well to each other.

There are also findings providing support for a three-factor structure. Lehto et al. (2003) used both exploratory and confirmatory factor analyses with 8 to 13 year-old-children, and identified three interrelated factors which had an approximate correspondence with EWM, inhibition and shifting. In addition, Wu et al. (2011) found that this three-factor structure of EF in individuals aged between 7 and 14 years also provided the best fit for their data.

Thus, in the primary school years, it is possible to identify separable factors involving EF abilities, but there is a lack of agreement about the composition of these factors. Most investigations have used confirmatory factor analysis to identify the factor structure that best fits the relevant data. Given the uncertainty about which model is supported by theory and previous research, we used exploratory factor analysis (EFA) rather than confirmatory factor analysis (CFA).

### Further Measures of EF in Children: Planning and Fluency

Planning and fluency are often studied in patients with frontal lobe damage and reflect a range processes that are relevant for everyday life (e.g., Pennington and Ozonoff, 1996). However, although these processes involve potentially important assessments of EF, there are uncertainties about how they relate to EWM, inhibition and shifting.

Our planning measure was the “sorting” task from the Delis–Kaplan Executive Functioning System (D-KEFS; Delis et al., 2001) and involved grouping cards into equal sized sets based on card features such as size, shape, and concept. According to the manual, this task assesses problem-solving, in particular concept-formation and rule generation. As with many EF tasks it may also assess inhibition of previous responses (Swanson, 2005), and more generally the task has been thought to assess planning ability (Henry et al., 2012). Furthermore, although planning is sometimes regarded as another component of EF, it also has been argued as being a higher order construct (Diamond, 2013). Research on the D-KFES Sorting task has been limited, but performance on the task appears to differentiate between children with disabilities and children with typical development (Mattson et al., 1999).

The other additional EF assessment concerned fluency, the ability to generate as many different examples of a class of items as possible within a short time period. The usual tasks used to assess verbal fluency involve target categories such as animals or words beginning with a particular letter (semantic and phonemic fluency, respectively); a common example of a non-verbal fluency task involves drawing as many different shapes as possible on a template of the same pattern of dots (design fluency). There are limited findings that fluency relates to some of the three commonly identified components of EF. For example, Lehto et al. (2003) reported that performance on semantic and phonemic fluency tasks was related to performance on a shifting task (Trail Making), while Rosen and Engle (1997) found that verbal fluency was related to working memory ability. There has also been discussion of whether fluency is more closely related to EF or language abilities (Shao et al., 2014; Henry et al., 2015; Whiteside et al., 2016; Marshall et al., 2017). Consequently, there is a need to understand the way that verbal and non-verbal fluency relate to the more usual assessment of EF.

### Relationships Between EF and Language Ability

Our interest in the structure of EF also concerned whether verbal and non-verbal assessments were grouped into separate factors. There has been discussion about whether EF is influenced by language ability or *vice versa* (Bishop et al., 2014). In two previous investigations findings indicated that the influence of language disorder on EF is not confined to verbal tasks, but also extends to non-verbal EF tasks, something that would not be expected if language disorders only had a direct and specific effect on tasks which involve verbal operations (Henry et al., 2012; Yang and Gray, 2017).

However, different findings have been reported about the relationships between language ability and EF in students who are deaf. These students often have delays in the progress of spoken and/or sign languages and this could affect verbal and non-verbal EF performance. In these investigations there is more evidence that language ability influences performance on EF tasks rather than *vice versa* (Figueras et al., 2008; Botting et al., 2016). Jones et al. (unpublished), using cross lagged regressions, confirmed that language led EF developmentally and not just at the performance level, although this effect was stronger for deaf children than hearing participants.

A further viewpoint is provided by Gooch et al. (2016) who failed to identify influences in either direction between EF and language in children at risk for dyslexia and typically developing children: the abilities appeared to develop together, but did not influence each other. This was interpreted as supporting the existence of a third influence, such as processing speed, on both EF and language, which causes relationships between the two domains.

Factor analyses provide an additional way to investigate these issues about language and EF by examining the relationships between non-verbal and verbal EF tasks. If language abilities only affect performance on verbal tasks and not non-verbal tasks, it might be expected that verbal EF tasks would be a notable feature of one factor, and that non-verbal EF tasks would be a notable feature of another factor. Such findings would provide additional indirect evidence about the relationship between language and EF.

### The Current Study

Our investigation of the factor structure of EF in the primary school years was carried out on data already collected from typically developing children in two previous studies (Henry et al., 2012; Leonard et al., 2015). The same assessments of EF were used in both investigations, and to ensure comparability in the measures, separate *z*-scores were calculated for each sample, which should minimize the effect of any confounds. The research was designed to address three research questions concerning children in the 6–12 year age range:

(1) Does EFA using verbal and non-verbal EF tasks assessing EWM, inhibition and switching produce a factor structure that is similar to one of those reported in previous investigations?
(2) Does the inclusion of fluency and planning assessments in the EFA analysis produce modifications to the initial factor structure?
(3) Is there evidence for language having an influence on the structural organization of verbal and non-verbal EF tasks?

## Materials and Methods

### Participants

A total of 159 participants were recruited to be part of the typically developing comparison groups of two investigations concerned with EF, one study was concerned with specific language impairment (SLI) and the other with developmental coordination disorder (DCD). The former study recruited 88 children with typical development and the latter 71 children with typical development; 14 children recruited into the SLI study were excluded to give an age range in the remaining sample between 6 and 12 years 6 months [SLI study mean age 9:2 years (*SD* 23 months); DCD study mean age 9:5 years (*SD* 12 months)].

The selection criteria in the two investigations ensured that children considered as typically developing in each study were distinguishable from the target clinical groups. Thus, both groups of children with typical development met acceptable, but slightly different, criteria for inclusion. In the SLI study the criteria for inclusion were non-verbal abilities in the average range as assessed by BAS-II Matrices (T-scores of 40 or greater, mean = 50, SD = 10; British Ability Scales-II, Elliott et al., 1996) and scaled scores of eight or more on four CELF-4-UK subscales (Clinical Evaluation of Language Fundamentals-4-UK; Semel et al., 2006; see below). In the DCD study, the inclusion criteria were a General Cognitive Index of 70 or above (calculated from BAS3, Word Definitions, Verbal Similarities and Matrices subscales; Elliot and Smith, 2011), together with at least one standard score of four or above on two CELF-4-UK subtests (Formulated Sentences and Word Classes-Receptive). The children in the latter study also had to have percentile scores equal to or above 25 on the Movement Assessment Battery for Children (MABC-2; Henderson et al., 2007) and a standardized score of 70 or above on the Test of Word Reading Efficiency (TOWRE; Torgesen et al., 1999).

To help to ensure comparability between the two samples, children from the DCD study were excluded if their Matrices subscale T-score was below 40 and if either of the two CELF-4-UK subscales administered were below eight. This excluded 17 children, so the remaining total sample consisted of 128 participants (mean age 111.13 months, *SD* 19.59; there were 58 female participants). The standardized scores from the BAS-II (SLI study) and BAS3 (DCD study) for verbal ability were SLI, 111.56 (*SD* 10.39) and DCD, 108.70 (*SD* 10.77). The T-scores for the BAS matrices assessment were, respectively, 52.03 (*SD* 6.29) and 52.63 (*SD* 8.19). The mean scores for both groups of children were slightly above average and this probably reflects the selection criteria for both these samples.

The children were recruited from schools within Greater London and, in the study involving children with SLI, very occasionally, via direct contact with parents/guardians. The catchment areas of the schools were variable in nature, but predominately low to mid socio-economic status. All the children were regarded by their assessors as having typical levels of spoken English and no child appeared to have English as a second language. All the children in the sample had BAS verbal standardized scores above 89.

For the study that concerned children with SLI, testing took place across 3–8 sessions, making up 3½ h for the complete battery, usually at school but occasionally at the child’s home. For the DCD study, 5–6 sessions of 45 min to 1 h each were conducted at school, making up 5 h for the complete battery. A range of non-EF assessments were also carried out in these investigations and further details about the general findings are described in our other publications (Henry et al., 2012; Leonard et al., 2015). Measures were administered in random orders to participants.

The projects were granted ethical approval from the appropriate University Research Ethics Committees, and were discussed in detail with relevant school staff before recruitment. Informed consent for participation was obtained in writing (telephone permission occasionally) from parents/guardians; children/students also gave their oral and written assent and were told they could opt out at any time.

### EF Tasks

Each executive ability was assessed using pairs of tests, one for the verbal domain and one for the non-verbal domain. We used various strategies to try to select comparable verbal and non-verbal tasks that assessed predominantly the construct in question. In some cases it was possible to use assessments which had the same task structure, but involved either verbal or non-verbal behavior (e.g., inhibition), in other cases we were guided by theoretical models which have resulted in different tasks to assess comparable verbal and non-verbal abilities (e.g., EWM), or we used similar tasks from the same assessment battery which involved either a verbal or non-verbal response (e.g., fluency and planning). Although, the tasks were selected to provide a useful test of differences between verbal and non-verbal functioning, we are not claiming that task purity was achieved.

#### Executive Working Memory

Executive working memory requires concurrent processing and storage. The verbal task was Listening Recall (Working Memory Test Battery for Children, WMTB-C; Pickering and Gathercole, 2001). A series of short sentences were read to the children and they judged whether each was true/false (processing). The children were then asked to recall the final word from each sentence in correct serial order (storage). The first trials had a list length of one item, and the task progressed on to longer lists, with six trials per list length, until 4/6 trials were incorrect. Total trials correct were scored. Test–retest reliabilities of 0.38–0.83 are reported for the relevant ages (Pickering and Gathercole, 2001).

The odd-one-out test was the non-verbal EWM task (Henry, 2001). The Experimenter presented three cards showing simple nonsense shapes (horizontally orientated on 20 cm × 4 cm cards). The child pointed to the shape which was the “odd-one-out” (processing). Storage was assessed via response sheets (20 cm × 30cm) which had three “empty” boxes that represented the cards, so the child could point to the location of each identified “odd-one-out.” The first trial had one item, and the task progressed on to longer lists, with three trials per list length, until 2/3 trials were incorrect. Total trials correct were scored. The span version of this task has a reliability of 0.80 (Henry, 2001).

#### Inhibition

The “Verbal Inhibition, Motor Inhibition” test (VIMI; Henry et al., 2012) was used. This task had two types of response: to copy the Experimenter; or to inhibit copying and produce an alternative response. For part A of the *verbal task*, the Experimenter said either “doll” or “car” and the participant was asked to repeat the same word (block 1). Next, in block 2, the child was expected to inhibit repeating the response: “If I say doll, you say car; and if I say car, you say doll.” Next there was a second “copy” block and a second “inhibit” block. Each of the four blocks had 20 trials. This entire sequence was repeated in part B, with new stimuli (“bus” and “drum”). In the *non-verbal motor task* the same format was followed, but words were replaced with hand actions. For part A, the action was a pointing finger versus a fist; for part B the action was a flat horizontal hand versus a flat vertical hand. The total number of errors made across parts A and B on each task was used as the measure of inhibition and was expressed as a negative score. Cronbach’s alpha, based on total error scores from parts A and B was 0.915 for the non-verbal task, and 0.727 for the verbal task.

#### Switching

It was difficult to obtain simple and comparable measures of switching that were in the verbal versus visuospatial domains, the two selected were the verbal trail making task (D-KEFS; Delis et al., 2001) and the non-verbal Intra/Extra Dimensional Set Shift test (Cambridge Neuropsychological Test Automated Battery; Cambridge Cognition, 2006). The Trail Making Task requires continual switching between two classes of item (easily nameable numbers and letters), whereas the Intra/Extra Dimensional Shift test required children to learn a rule to guide responding and then switch to another rule unpredictably, and this task concerned stimuli that were not easily nameable. These are not identical tasks, but they both required children to switch between response sets and also required them to be flexible when responding. These tasks (and other similar versions of them) have been commonly used in previous literature to assess switching in both children and adults so have considerable face validity for measuring this construct.

In the Trail Making Test children joined small circles containing letters and numbers alternately, in sequence (1-A-2-B-3-C through 16-P). Four control conditions assessed component skills. The most relevant were: number sequencing (connecting numbers 1–16); and letter sequencing (connecting letters A–P). “Switching cost” was the total time taken for combined letter/number switching, minus the sum of the time taken for the number and letter sequencing component skills. These scores were multiplied by -1 so that as the scores increased from negative to positive this represented increasing switching ability. The letter sequencing and the number sequencing tasks were terminated after 150 s; the number–letter switching task was terminated after 240 s. Test–retest reliabilities for measures contributing to “switching cost” are reported as: number sequencing (0.77), letter sequencing (0.57); letter/number switching (0.20; Delis et al., 2001). Reliability for switching measures can be low, given they are difference scores; consequently, somewhat lower reliabilities are likely in this area (Henry and Bettenay, 2010).

For the intra/extra dimensional set shift task, initially, two colored stimuli were presented on a screen, and by touching one, the child could learn a rule from feedback about which was “correct.” Later, a second dimension, an irrelevant white line, was introduced. This introduced new stimuli, yet the child still needed to respond to the shape stimuli. The complex stimuli were later changed and the child had to switch attention to the previously irrelevant dimension to obtain “correct” responses (“extradimensional” shift). Total error scores were used (test–retest reliability reported as 0.40; Cambridge Cognition, 2006) and the scores were multiplied by -1 so that as the scores became less negative this represented increasing switching ability.

#### Fluency

Verbal fluency (D-KEFS; Delis et al., 2001) involved several versions of a similar task. In all tasks, the children were asked to say as many words as possible in 1 min according to a criterion. “Letter fluency” involved the letters F, A, and S; “category fluency” concerned the semantic categories of “animals” and “boys” names’. Verbal fluency was the total raw score from all five tasks.

Non-verbal fluency (Design Fluency, D-KEFS) involved a response booklet containing patterns of dots in boxes. The children were asked to draw as many different designs as possible in 1 min, each in a different box, by connecting dots with four straight lines (with no line drawn in isolation). Condition 1 consisted of only filled dots; Condition 2 consisted of arrays of filled and empty dots and the child connected only empty dots. Design fluency was the total raw score from these two conditions. Test–retest reliabilities are reported as: letter (0.67); category (0.70); filled dots (0.66); empty dots (0.43) (Delis et al., 2001).

#### Planning

The Sorting Test (D-KEFS) assessed verbal and non-verbal planning. Children sorted sets of six cards into two groups of three, in as many different ways as they could. There were three possible “verbal” sorts (e.g., transport/animals; things that fly/thing that move along the ground); and five possible “perceptual” sorts (e.g., small/large; straight/curved edges). Total numbers of correct verbal or perceptual sorts were used as the measures of verbal or non-verbal planning, respectively (test–retest reliability reported as 0.49; Delis et al., 2001).

## Results

The mean scores on the ten EF assessments are shown in **Table 1**. Bivariate correlations between the assessments are given in **Table 2** and show moderate correlations between variables, with no correlations above 0.50.

**Table 1:** Mean and standard deviations of the EF assessments.

Task	Task	Mean (*SD*) Raw score	Min to Max
**EWM**			
Verbal	Listening span (WMTB-C)	13.92 (3.43)	5 to 27
Non-verbal	Odd-one-out	9.98 (3.16)	4 to 17
**Fluency**			
Verbal	Verbal fluency (D-KEFS)	59.51 (14.25)	29 to 102
Non-verbal	Design fluency (D-KEFS)	14.76 (4.65)	4 to 27
**Planning**			
Verbal	Sorting task (D-KEFS)	2.50 (1.12)	0 to 5
Non-verbal	Sorting task (D-KEFS)	5.56 (2.15)	0 to 9
**Inhibition**			
Verbal	VIMI test^∗^	-8.24 (5.51)	-23 to 0
Non-verbal	VIMI test^∗^	-23.63 (12.28)	-59 to -5
**Switching**			
Verbal	Trail making test (D-KEFS)	-28.02 (32.32)	-132 to 60
Non-verbal	Intra/Extra dimensional shift (CANTAB)	-26.57 (11.22)	-55 to -8

*^∗^Verbal Inhibition, Motor Inhibition Test.*

**Table 2:** Bivariate correlations between EF assessments.

	EWM Verbal	EWM Non-verbal	Fluency verbal	Fluency verbal	Plan verbal	Plan Non-verbal	Inhib verbal	Inhib Non-verbal	Switch verbal
**EWM**									
Non-verbal	0.44								
**Fluency**									
Verbal	0.52	0.33							
Non-verbal	0.30	0.31	0.44						
**Planning**									
Verbal	0.24	0.18	0.27	0.33					
Non-verbal	0.31	0.20	0.30	0.29	-0.10				
**Inhibition**									
Verbal	0.12	0.25	0.09	0.04	-0.03	0.19			
Non-verbal	0.14	0.32	0.05	0.10	-0.05	0.11	0.42		
**Switching**									
Verbal	0.30	0.23	0.18	0.19	-0.12	0.22	0.16	0.07	
Non-verbal	0.26	0.19	0.36	0.11	0.05	0.32	0.20	0.10	0.17

To ensure comparability of data from the two samples, *z*-scores were calculated for each measure; this was done separately for each of the two samples and then the data were combined. This ensured that any differences due to sampling would be minimized. Examination of skewness and kurtosis was carried out, using a critical value for medium sized samples of 3.29 (Kim, 2013). The skewness and kurtosis of all the variables was acceptable except for the skewness of verbal working memory and verbal inhibition, and the kurtosis of verbal working memory. Inspection of the relevant graphs was carried out and they appeared acceptable given that univariate assumption of normality is not always considered as critical to factor analysis. Checks were made on univariate outliers and there were no extreme scores according to SPSS box plots. Mahalanobis distance was also checked and there was only one instance of a multivariate outlier, removal of this case did not influence the analyses.

Exploratory factor analysis (Principal Axis Factoring in SPSS) was used rather than CFA, as previous theory and research has produced different models of EF structures and we were limited to two variables for each construct. For the EFA analyses, Oblique rotation (oblimax) was employed, as it was thought that EF factors could be related to one another as suggested by the idea of unity and diversity (Miyake and Friedman, 2012). To check whether a different method of extraction and rotation resulted in different factors, principal components analyses (PCA) with orthogonal rotation (varimax) were also conducted. PCA is usually recommended for the derivation of scores rather than the investigation of factor structure, and varimax rotation is usually regarded as maximizing the spread of loadings within factors (Field, 2009). Consequently, the main interest was in the findings from the EFA, with the PCA analysis being used to check that a different form of analysis produced similar findings.

For the first analysis on the six core EF variables (i.e., EWM, inhibition, and switching), Bartlett’s Test of Sphericity was significant at *p* < 0.001 (95.67, *df* 15). The Kaiser-Meyer-Olkin statistic of sampling accuracy was 0.66, which is acceptable according to Tabachnick and Fidell (2013). Even so, caution should be exercised when interpreting the findings about the separation of variables into factors. The measures of sampling adequacy of the variables from the diagonals of the anti-image correlation matrix were all above 0.6 (for switching 0.74 and for the remaining variables between 0.61 and 0.68), and therefore were adequate (Field, 2009).

Two factors were identified by the analysis. The eigenvalues for the first three factors were: 2.1, 1.1, and 0.9 showing a reasonable separation between factors 2 and 3 which supports the choice of factors with eigenvalues above 1. The first two factors accounted for 54.86% of the variance. **Table 3** displays the pattern matrix (i.e., rotated) which provides information about the regression coefficients for each variable. Coefficients or loadings above 0.30 are displayed in this and the other table. The findings in the pattern matrix indicates that the first factor had the most important contribution from verbal EWM, and included non-verbal EWM as well as smaller contributions from the two switching variables. The second factor contained the two inhibition variables. This suggests the presence of two factors, one which primarily involved EWM and switching, and a second factor than involved inhibition. The organization of the variables into factors showed no evidence of a separation into verbal and non-verbal variables. The findings from the PCA analysis are also provided in **Table 3**. The major differences between the EFA and the PCA involve higher loadings from the PCA, which is often the case. Furthermore in the PCA, non-verbal working memory was identified with a loading of above 0.30 on the second factor involving inhibition.

**Table 3:** Pattern matrix for exploratory factor analyses (EFA; oblique rotation) and principal components analyses (PCA; varimax) on assessments of EWM, switching, and inhibition.

	EFA		PCA	
	Factor 1	Factor 2	Component 1	Component 2
**EWM**				
Verbal	0.86		0.80	
Non-verbal	0.49		0.66	0.41
**Switching**				
Verbal	0.40		0.60	
Non-verbal	0.32		0.54	
**Inhibition**				
Verbal		0.60		0.85
Non-verbal		0.70		0.80

For the analyses on the 10 EF variables (i.e., including verbal and non-verbal fluency and planning in addition to the six core EF variables) different structures were produced for the initial EFA and PCA analyses. These differences were only present when the verbal planning variable was entered into the analyses of the ten variables. There were other problematic issues with this variable. Verbal planning had the most limited range of scores of any variable and had the lowest measure of sampling adequacy in the anti-image correlation table. In addition, non-verbal planning which involved a very similar task, but with a greater range of scores, did not have the same problems. Consequently, it was decided to remove verbal planning from the analyses.

In the analyses of the nine EF variables (**Table 4**), the Kaiser-Meyer-Olkin statistic was acceptable (0.74) as was Bartlett’s test of sphericity (208.85, *p* < 0.001, *df* 36). The measures of sampling adequacy figures also were acceptable, as all were above 0.62 (verbal and non-verbal inhibition 0.62–69; verbal switching was 0.82, and the remaining variables were between 0.70 and 0.79). In the analysis using EFA with oblique rotation, two factors were identified and the eigenvalues for the first three factors were: 2.9, 1.4, and 1.0 showing a reasonable separation between factors 2 and 3. The first two factors accounted for 47.56% of the variance in total, 32.45 and 15.11%, respectively.

**Table 4:** Pattern matrix for exploratory factor analyses (EFA; oblique rotation) and principal components analyses (PCA; varimax) on assessments of EWM, switching, inhibition, fluency, and non-verbal planning.

	EFA		PCA	
	Factor 1	Factor 2	Component 1	Component 2
**EWM**				
Verbal	0.69		0.74	
Non-verbal	0.46		0.53	0.43
**Switching**				
Verbal	0.34		0.45	
Non-verbal	0.40		0.50	
**Fluency**				
Verbal	0.80		0.79	
Non-verbal	0.54		0.65	
**Planning**				
Non-verbal	0.47		0.58	
**Inhibition**				
Verbal		0.66		0.81
Non-verbal		0.64		0.82

The pattern matrix reported in **Table 4** shows that the majority of the variables contributed to the first factor, with the most important contributions from verbal fluency and verbal EWM. The second factor was made up of verbal and non-verbal inhibition. The findings did not show an obvious separation of variables according to whether or not they involved verbal or non-verbal EF tasks.

A further analysis on the same variables conducted using PCA with varimax rotation is also reported in **Table 4**. The findings were similar to the EFA in that all the variables except for verbal and non-verbal inhibition loaded on the first factor, the most notable difference to the EFA analysis was that verbal working memory had a low loading on factor 2. Again, verbal working memory and verbal fluency made the largest contributions to component 1.

## Discussion

### The Structure of EF in Primary School Aged Children

Exploratory factor analyses and PCAs were conducted on data concerning verbal and non-verbal assessments of EF obtained from 128 typically developing children aged between 6 and 12 years. The findings from an EFA involving the core EF tasks of EWM, inhibition, and switching identified two factors. The first factor had contributions from all the four EWM and switching variables, and the second factor consisted of verbal and non-verbal inhibition. A PCA produced similar findings, although in this case there was evidence from the component loadings of weak links between non-verbal EWM and inhibition.

Further analyses were conducted with the inclusion of verbal and non-verbal, planning and fluency. The initial analyses indicated that the inclusion of verbal planning resulted in different structures in EFA and PCAs. Because these two sets of analyses are usually expected to produce similar findings, and verbal planning had poor psychometric properties, it was decided to remove the verbal planning variable from subsequent analyses. Further EFA on the nine remaining EF variables resulted in a two-factor solution. The first factor had contributions from verbal and non-verbal EWM, verbal and non-verbal switching, verbal and non-verbal fluency, and non-verbal planning. The second factor was made up of verbal and non-verbal inhibition. The PCA produced similar findings, and again there was a weak contribution from non-verbal EWM to the inhibition factor. Consequently, the additional fluency and planning variables loaded onto the first factor/component in both analyses, which appeared to involve a general EF ability. It was notable that both verbal EWM and verbal fluency had the highest loadings on this factor.

The analyses on the nine variables using different forms of data reduction produced very similar outcomes, however, it needs to be acknowledged that this only occurred after excluding verbal planning from the analyses. This variable had a low range of scores and a low measure of statistical adequacy, which provided a justification for its removal. In addition, non-verbal planning which involved very similar activities, but had a greater range of scores, did not have the same problems. Consequently, although there are advantages of the D-KEFS assessment of verbal planning, as it seems less affected by the task impurity problems associated with Tower tasks, it may have disadvantages when used with children between 6 and 12 years. Future research might consider alternative assessments of verbal planning with better psychometric properties and less restricted variance. More generally, it also would be desirable to have a greater number of assessments for each construct and a larger sample size than in this investigation.

Thus, the current analyses provided support for an inhibition factor and a general EF factor involving EWM, switching, fluency, and planning. The findings are consistent with previous research in children between 6 and 12 years as more than one EF factor was identified. However, previous research has largely considered only three EF components, namely EWM, switching, and inhibition. A novel contribution of the current study is that adding measures of planning (non-verbal) and fluency (verbal and non-verbal) resulted in the same two-factor structure, with the additional measures loading largely on a general EF factor. In relation to these findings, it is worth noting that factor analysis is less effective than structural equation modeling with a larger sample in identify whether planning, as has been previously discussed (Diamond, 2013), is a higher order EF structure.

### Explanations for Different EF Structures

One general issue in relation to our findings concerns the reasons why two-factor solutions should be the most common description of the organization of EF between 6 and 12 years. Part of the answer is likely to be that the period between 6 and 12 years represents a progression from the one-factor solutions that are reported at younger ages (Wiebe et al., 2008, 2011; Hughes et al., 2010) before reaching the more complex three-factor solutions identified in adulthood (Miyake and Friedman, 2012). The one-factor solutions reported in pre-school children suggest that individual differences in EF abilities are similar across all aspects of EF. This may be the result of a set of general problem-solving abilities, such as core components relating to self-control or self-regulation (e.g., Miyake and Friedman, 2012), attentional abilities (Garon et al., 2008) or processing speed (Gooch et al., 2016) influencing performance across a wide range of EF tasks, with the result being consistent individual differences across the different EF tasks.

The commonly reported finding of a two-factor EF structure during the primary school years has been replicated here, and suggests that during this age range more specialist and differentiated mental capacities are available. In terms of individual differences, this implies that some children become good at one aspect of EF while other children become good at another. However, this development should not result in the variability we see in factor structures across different investigations. For example, in previous research, there is more evidence for a separation into abilities which are relevant to updating/EWM on the one hand, and inhibition-switching abilities on the other, as suggested by Lee et al. (2013). Nevertheless, there are also reports of a separation into abilities relevant to inhibition versus EWM-switching abilities, as suggested by St Clair-Thompson and Gathercole (2006), mirroring the findings of our analyses.

It is possible that the different factor structures in the 6–12 years age range are a product of task impurity (Miyake et al., 2000). It is generally agreed that task impurities result in performance on assessments being driven by several different EF abilities and potentially other non-EF abilities (Friedman et al., 2008). Across different investigations, task impurity could mean that even different tasks believed to assess the same EF component may have different relationships with other EF tasks. CFA analysis with the use of latent variables helps to avoid this type of problem (Miyake and Friedman, 2012), but even here the latent variable will be dependent on which tasks have been chosen to represent it. Consequently, if different investigations use different tasks to assess each of the three EF components, this is likely to result in different factor structures across the investigations. It is possible that a larger number of tasks to assess each component of EF ability and a larger number of children would result in greater consistency, but ethical and practical constraints on testing time and participant numbers make it extremely difficult to achieve this.

Not only is task impurity an issue, but a related problem is that there is variation between investigations about which tasks assess the most relevant characteristics of an EF component. For example, a range of tasks have been used to provide indicators of inhibition ability, and the use of very similar inhibition tasks is likely to result in a more coherent and stronger underlying factor or latent variable. In our study the two assessments of inhibition had very similar task demands and inhibition was identified as a separate factor. In contrast, Huizinga et al. (2006) could not identify a common factor from the three different assessments of inhibition that they used (specifically, stop signal, flanker, and stroop). These issues about the choice of variables that are entered into a factor analysis may be as important as some of the statistical considerations in determining the factor structure, but it is much more difficult to specify what is best practice.

A further reason for different factor structures across investigations is that our conceptualization of the identity of the different forms of EF ability in the 6–12 age range needs to be further refined. Much of the thinking about the components of EF appears to be task-based and this is a sensible initial approach. However, we may need to consider potential neurocognitive processes that give rise to different EF abilities (Anderson, 2002), and so take a more brain-orientated and cognitive-based approach to the abilities underlying EF. This could involve investigating the brain structures which are activated during different EF tasks and using this as a basis to help identify those areas which are common to different EF processes.

### Language and EF Abilities

If we had found that verbal and non-verbal EF tasks loaded on different factors, this would have provided strong support for the idea that verbal ability has an influence on verbal EF tasks. However, the factors that were identified contained a mix of verbal and non-verbal variables. Consequently, the findings from this study failed to provide support for the argument that language ability directly affects verbal EF abilities at the task performance level (Bishop et al., 2014).

Although, these findings are consistent with the idea that language ability is not an important influence on EF performance, our evidence in support of this position is limited in nature, especially as there is a range of sources of evidence that should be used to address this complex question (Botting et al., 2016). In other words, our data are not able to provide clear support for the idea that language does not influence EF abilities. This is because the evidence is cross-sectional, correlational in nature and consists of the absence of a positive effect. Further, we acknowledge that the relationship between language and EF abilities is complicated by the fact that verbal abilities are relevant to non-verbal tasks in order to understand instructions, and for the operation of inner speech which could be utilized during EF tasks; it also might be that some non-verbal processes have an influence on verbal tasks (e.g., certain forms of inhibition). Thus, the current findings do not provide definitive evidence about the relationship between EF and language. Rather, they provide support for the idea that concurrent language ability does not differentially affect performance on tasks selected to assess verbal and non-verbal EF.

### Summary

Our findings support previous research concerning two-factor structures of EF in the primary school years, and suggest that planning and fluency contribute to a general EF factor. However, the current findings and those from previous investigations about the *composition* of the factors suggest that future research should keep in mind important methodological considerations relating to EF measures, and that task influences may be as important as individual differences in determining factor structures. Our findings did not provide evidence of separable verbal and non-verbal factors, and consequently failed to provide support for an effect of language ability on EF. Finally, research and theorizing could benefit from a greater focus on basic neurocognitive operations that underlie performance on EF tasks, to more fully understand the developmental, clinical, and educational implications of differentiation in EF with age.

## Data Availability

The raw data supporting the conclusions of this manuscript will be made available by the authors, without undue reservation, to any qualified researcher.

## Author Contributions

DM led on the writing and analyses. MB contributed to writing, analyses, and data collection. LH was principal investigator on both studies, the remaining authors made equivalent contributions to writing and/or data collection.

## Conflict of Interest Statement

The authors declare that the research was conducted in the absence of any commercial or financial relationships that could be construed as a potential conflict of interest.
